# Evaluation of intensity‐modulated radiation therapy factors by treatment site and technique for various linear accelerators

**DOI:** 10.1002/acm2.70200

**Published:** 2025-08-21

**Authors:** Jin Jegal, Min‐Jae Park, Sang Hoon Jung, Sungkoo Cho, Jungwon Kwak, Jung‐in Kim, Chang Heon Choi

**Affiliations:** ^1^ Department of Radiation Oncology Seoul National University Hospital Seoul Republic of Korea; ^2^ Institute of Radiation Medicine Seoul National University Medical Research Center Seoul Republic of Korea; ^3^ Department of Radiation Oncology, Asan Medical Center University of Ulsan College of Medicine Seoul Republic of Korea; ^4^ Department of Radiation Oncology Samsung Medical Center Seoul Republic of Korea; ^5^ Biomedical Research Institute Seoul National University Hospital Seoul Republic of Korea; ^6^ Department of Radiation Oncology Seoul National University College of Medicine Seoul Republic of Korea

**Keywords:** IMRT factor, LINAC, VMAT

## Abstract

**Purpose:**

The intensity‐modulated radiation therapy (IMRT) factor, which is essential for calculating leakage radiation and secondary barrier thicknesses, is represented by the ratio of average monitor units (MUs) per unit dose in IMRT compared to conventional radiotherapy. The National Council on Radiation Protection and Measurements recommends a broad range of 2–10 for this factor, which can complicate its practical use. This study aims to calculate and compare the IMRT factors of Halcyon, VitalBeam, and TrueBeam based on actual MUs, offering a more accurate foundation for effective and reasonable radiation shielding determination.

**Methods:**

Treatment data from three hospitals in Korea, collected between March 25, 2022, and December 31, 2023, was extracted using the ARIA Unified Reports Application. IMRT factors were calculated by analyzing actual MUs in fixed‐gantry IMRT, volumetric modulated arc therapy (VMAT), and stereotactic ablative radiotherapy techniques across three different linear accelerators (LINACs) by treatment sites.

**Results:**

Based on the analysis of actual treatment records, the average IMRT factor for the VMAT technique was calculated as 2.82 ± 0.32 for Halcyon, 2.61 ± 0.31 for TrueBeam, and 2.22 ± 0.21 for VitalBeam, indicating that the IMRT factor for Halcyon was approximately 8% higher than TrueBeam and 27% higher than VitalBeam. Although Halcyon exhibited a tendency to use more MUs, the difference was not significant enough to warrant applying the IMRT factor of 10. The IMRT factor for fixed‐gantry IMRT on Halcyon was about 1.9 times greater than that for its VMAT technique. No significant variation was observed across treatment sites.

**Conclusions:**

The presented values may assist in evaluating secondary leakage radiation and contribute to improving the practicality and efficiency of radiation shielding design by providing reference data grounded in actual clinical treatment records.

## 1 INTRODUCTION

Intensity‐modulated radiation therapy (IMRT) has been established as a standard and optimal technique for tumor treatment.^1^, ^2^ It has the advantage of creating various dose‐fluence maps in treatment regions while sparing organs at risk by optimizing multi‐leaf collimator (MLC) modulation. However, because MLC leaves create many beamlets and small fields during modulation, the total accelerator monitor units (MU) required are much higher than those required for conventional radiotherapy (RT) to deliver the same absorbed dose to the patient.^3^, ^4^ These differences are represented by the IMRT factor, which is the ratio of the average MU per unit prescribed absorbed dose in IMRT treatment to that in conventional RT. The IMRT factor contributes to the leakage radiation workload and may affect the total dose rate by requiring either extended beam‐on time or adjustments to the dose rate settings to maintain treatment efficiency.

Currently, the IMRT factor presented by the National Council on Radiation Protection and Measurements (NCRP) 151 varies widely, ranging from 2 to 10 (except for CyberKnife vaults).^3^ The Halcyon linear accelerator (LINAC) by Varian Medical Systems (Palo Alto, CA) is reported to require twice the monitor units (MUs) compared to the Varian TrueBeam to deliver a prescribed dose, with the TrueBeam using up to 5 MUs per cGy for IMRT.^5^ With the growing adoption of Halcyon, accurately determining the IMRT factor has become increasingly important. If workload assumptions are based on an inaccurately predicted IMRT factor, this could lead to the conclusion that a higher MUs per cGy is required compared to other LINACs. From a clinical management perspective, if the IMRT factor is overestimated, the workload will increase, potentially exceeding the weekly radiation dose limits set for the shielding facility. This may restrict the number of patients that can be treated. Additionally, if adjustments to the shielding design are required, such as reinforcing the shielding walls, it could result in additional construction costs.

In this study, we calculated the IMRT factors based on treatment data with Halcyon, VitalBeam, and TrueBeam LINACs at three hospitals in Korea using the ARIA Unified Reports Application (AURA). AURA leverages the Varian system database to create and update unified report data models containing denormalized tables with attributes essential for constructing up‐to‐date and comprehensive reports. These data models, along with Microsoft Report Builder (an included module in the Microsoft SQL Server Business Intelligence Development Studio), allow writing direct queries for reports. By utilizing AURA, we efficiently collected and computed MUs across all fractions based on real‐time data, bypassing the cumbersome process of individually analyzing each patient's Dicom Plan file. The design and specifications of the MLC system can affect the beam, leading to variations in optimized total MUs and IMRT factors. Therefore, these three LINACs were compared.

## 2 METHODS

### 2.1 Treatment information collection

Treatment data were collected from three hospitals in Korea between March 25, 2022, and December 31, 2023. All treatment methods except fixed‐gantry IMRT utilized VMAT. Treatment with five or fewer fractions was classified as stereotactic ablative radiotherapy (SABR). The number of plans performed for each treatment site is summarized in Table 1. Data regarding the treatment site, MUs, and prescription doses for each plan were collected.

**TABLE 1:** Number of plans by LINAC and treatment site.

Treatment site		Lung	Breast	Chest	Abdomen	Cervix	Pelvis	Ext.	Prostate	Spine	Rectum	H/N	Brain
Halcyon	VMAT	311	1801	786	429	44	739	64	898	728	458	773	379
	SABR	1	–	5	16	–	2	–	1	7	–	–	–
	Fixed‐ gantry IMRT	234	3	252	21	–	349	19	22	257	156	97	1
TrueBeam	VMAT	781	1017	467	611	185	387	100	15	312	433	870	342
	SABR	251	2	45	87	5	81	27	11	232	2	36	–
	Fixed‐gantry IMRT	–	–	1	–	–	–	–	–	–	–	–	–
VitalBeam	VMAT	87	1392	229	69	25	87	25	31	95	24	165	10
	SABR	–	–	–	–	–	–	–	–	1	–	–	–
	Fixed‐gantry IMRT	–	–	–	–	–	–	2	–	–	–	–	–

Abbreviations: IMRT, intensity‐modulated radiation therapy; LINACs, linear accelerators; SABR: Stereotactic ablative radiotherapy; VMAT: volumetric modulated arc therapy.

* Ext.: Extremity and H/N: Head and neck.

### 2.2 ARIA unified reports application (AURA)

**FIGURE 1:**
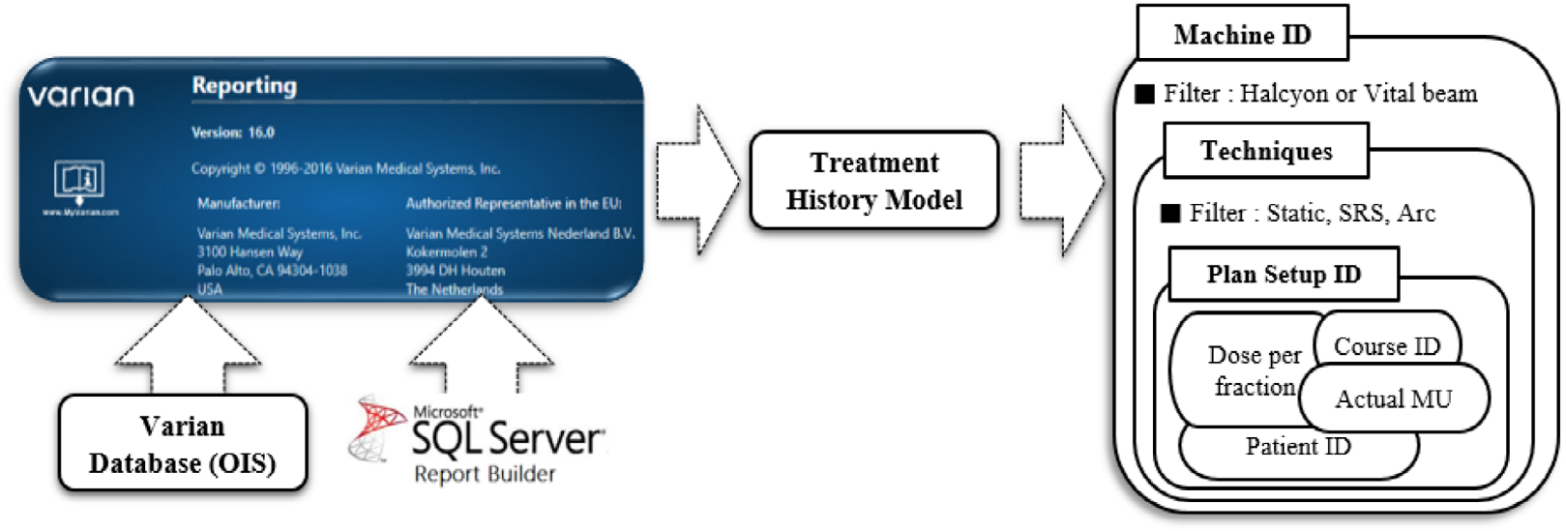
A flowchart of AURA. The AURA is connected to the Varian database, an oncology information system, with SQL server integration for data management. Through SQL, AURA accesses the Treatment History Model Dataset, allowing for variable filtering and selection for further analysis. AURA, ARIA unified reports application.

All data was retrieved and aggregated using AURA. We extracted treatment time, patient ID, actual MU, and prescription dose for each plan course as shown in Figure 1. Through post‐processing of the data, we classified the course IDs into 12 different treatment sites. We utilized Python 3.7 to classify the data by including the common substring of the course ID of the plan and the treatment site name.

### 2.3 Calculations

The IMRT factor was calculated using the following formula with the conventional IMRT factor (*C_I_*), denoted as *MU_CONV_* of 1.2 when using a 6 MV beam, as referenced in:^6^

$$C_{I}=\frac{MU_{\mathrm{IMRT}}}{MU_{\mathrm{CONV}}}$$

where *MU*_IMRT_ is the average monitor unit per unit of the prescribed dose absorbed by the patient, calculated as follows:^3^

$$MU_{\mathrm{IMRT}}=\sum_{i}\frac{MU_{i}}{{\left(D_{\mathrm{pre}}\right)}_{i}}$$

where *D*_pre_ is the unit prescribed absorbed dose per fraction and *MU_i_* is the total MU required to deliver a *D*_pre_ for *i* cases. *C_i_* is the IMRT factor, which is equal to *MU*_IMRT_ by *MU*_CONV_.

## 3 RESULTS

**FIGURE 2:**
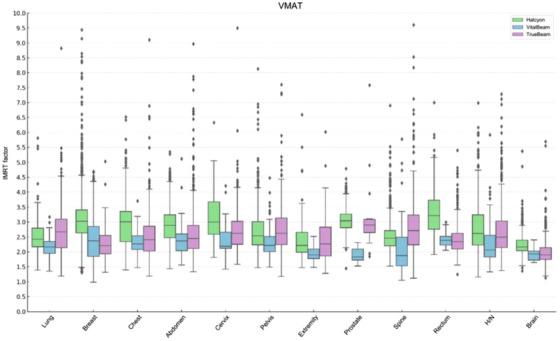
IMRT factor distribution in volumetric modulated arc therapy technique by linear accelerators and treatment site. IMRT, Intensity‐modulated radiation therapy.

The IMRT factors for each LINAC and treatment site were calculated and categorized according to VMAT, SABR, and fixed‐gantry IMRT techniques. Since there were no comparison groups other than the VMAT technique, the distribution of IMRT factor values for each equipment and treatment site is illustrated in Figure 2. Fixed‐gantry IMRT technique specifically refers to the use of static fields in the treatment plan. Both of VMAT and SABR techniques demonstrated that the differences among the three LINACs were not significant. However, the fixed‐gantry IMRT technique showed significantly higher MUs, with the Halcyon system consuming 1.9 times higher MUs compared to the VMAT technique. The mean values calculated for each boxplot, along with the data spread determined using the standard deviation from the mean, are summarized in Tables 2, 3, 4. The average IMRT factors for the VMAT technique were calculated to be 2.82 ± 0.32 for Halcyon, 2.61 ± 0.31 for TrueBeam, and 2.22 ± 0.21 for VitalBeam.

**TABLE 2:** The IMRT factor mean and standard deviation for VMAT by LINACs.

Technique	VMAT											
Treatment site	Lung	Breast	Chest	Abdomen	Cervix	Pelvis	Ext.	Prostate	Spine	Rectum	H/N	Brain
Halcyon	2.53 ± 0.54	3.08 ± 0.87	2.93 ± 0.72	2.93 ± 0.63	3.20 ± 0.90	2.71 ± 0.73	2.48 ± 0.92	3.05 ± 0.38	2.56 ± 0.57	3.29 ± 0.71	2.82 ± 0.84	2.27 ± 0.45
TrueBeam	2.66 ± 0.70	2.26 ± 0.44	2.51 ± 0.78	2.64 ± 0.92	2.78 ± 0.88	2.79 ± 0.83	2.40 ± 0.79	3.22 ± 1.37	2.93 ± 1.13	2.45 ± 0.56	2.69 ± 0.81	2.03 ± 0.52
VitalBeam	2.15 ± 0.33	2.45 ± 0.72	2.31 ± 0.35	2.42 ± 0.55	2.45 ± 0.62	2.35 ± 0.53	1.93 ± 0.27	1.89 ± 0.23	2.11 ± 0.83	2.42 ± 0.24	2.24 ± 0.65	1.93 ± 0.23

Abbreviations: Ext., extremity; H/N: head and neck; VMAT, Volumetric modulated arc therapy.

**TABLE 3:** The IMRT factor mean and standard deviation for SABR by LINACs.

Technique	SABR										
Treatment site	Lung	Breast	Chest	Abdomen	Cervix	Pelvis	Ext.	Prostate	Spine	Rectum	H/N
Halcyon	4.10	–	2.26 ± 0.71	3.74 ± 0.39	–	3.32 ± 0.04	–	2.29	3.47 ± 0.92	–	–
TrueBeam	2.38 ± 0.44	3.24 ± 0.74	2.24 ± 0.58	2.55 ± 0.66	2.61 ± 0.31	2.59 ± 0.80	2.30 ± 1.04	2.75 ± 0.49	2.97 ± 0.70	2.82 ± 0.61	2.22 ± 0.38
VitalBeam	–	–	–	–	–	–	–	–	3.31	–	–

Abbreviations: Ext.: extremity, H/N, Head and neck; SABR, stereotactic ablative radiotherapy;.

**TABLE 4:** The IMRT factor mean and standard deviation for fixed‐gantry IMRT by LINACs.

Technique	Fixed‐gantry IMRT											
Treatment site	Lung	Breast	Chest	Abdomen	Cervix	Pelvis	Ext.	Prostate	Spine	Rectum	H/N	Brain
Halcyon	4.68 ± 1.44	4.89 ± 0.59	4.59 ± 1.39	5.41 ± 1.74	–	6.33 ± 2.13	3.81 ± 0.91	7.10 ± 0.89	4.13 ± 1.16	6.00 ± 1.37	5.95 ± 1.83	5.94
TrueBeam	–	–	4.23	–	–	–	–	–	–	–	–	
VitalBeam	–	–	–	–	–	–	4.86 ± 0.58	–	–	–	–	

Abbreviations: Ext. extremity; H/N, head and neck; IMRT, intensity‐modulated radiation therapy.

**FIGURE 3:**
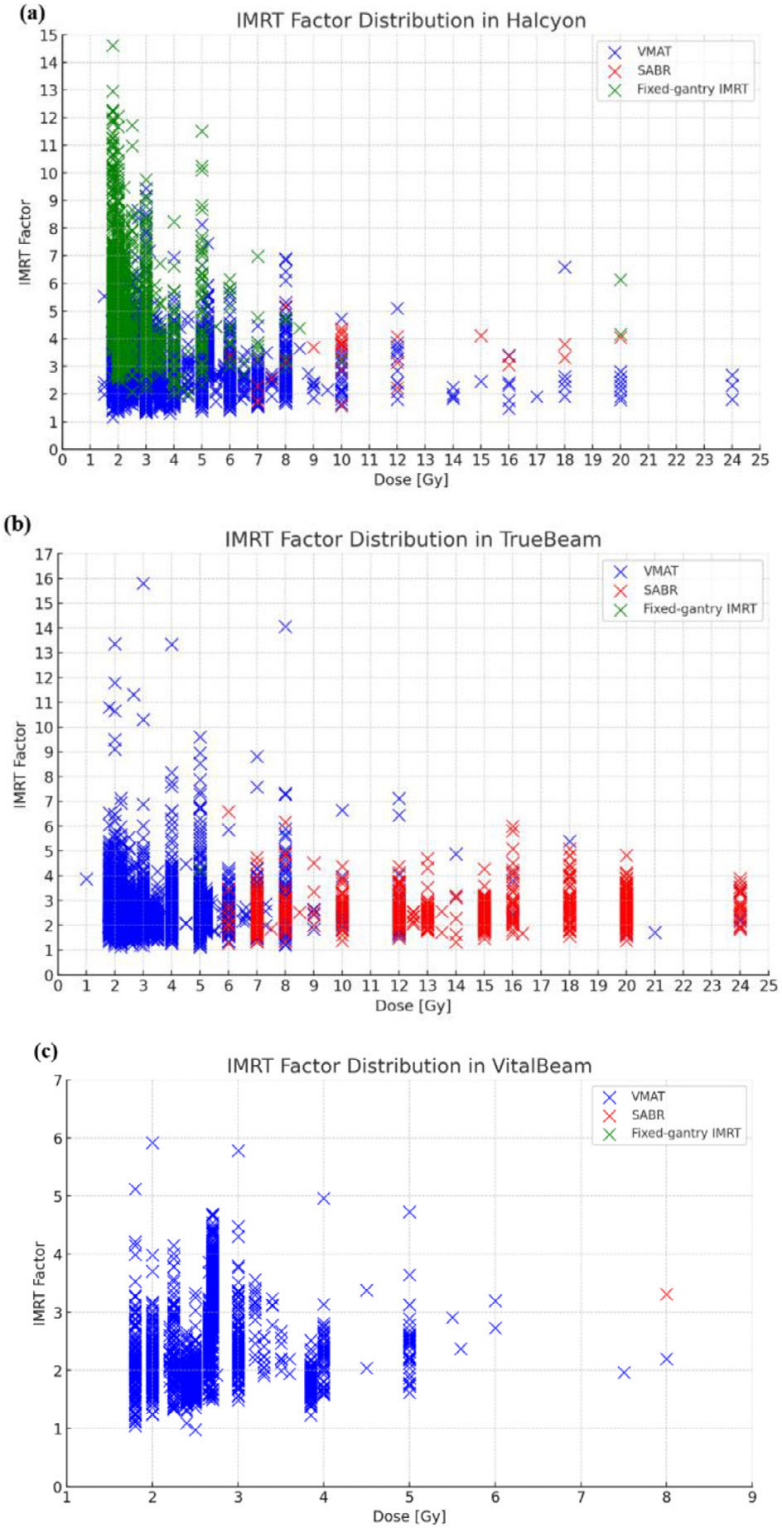
IMRT factor distributions of (a) Halcyon, (b) TrueBeam, and (c) VitalBeam according to prescription dose.

The distributions of the IMRT factors according to the techniques and prescription doses for each LINAC were analyzed. For all techniques and LINACs, no linear correlation with the prescription dose was observed. Similar to other techniques, SABR showed comparable IMRT factor values corresponding to the prescription dose (Figure 3). The number of SABR treatments was only valid for TrueBeam (Figure 3b).

## 4 DISCUSSION

The advancement and widespread application of dynamic MLCs have led to the predominance of IMRT techniques. Fixed‐gantry IMRT utilizes multiple fixed radiation beams at different angles to deliver radiation to the treatment area. VMAT is an evolved form of fixed‐gantry IMRT, which continuously delivers radiation while rotating around the treatment area. The VMAT technique modulates MLC, dose rates and gantry speeds during rotation, allowing for the creation of complex dose distributions.^7^ Compared to conventional RT techniques like 3D‐CRT, the use of dynamic MLCs to shape fields results in higher MUs.^4^, ^8^

As the number of MUs increased with each treatment, the IMRT factor, which was multiplied by the IMRT workload value, subsequently increased the leakage radiation workload.^3^ This decreases the leakage transmission factor, necessitating more tenth‐value layers for shielding. In clinical practice, shielding designs are already conservatively planned by accounting for various uncertainties in actual construction materials (elemental constituents of concrete, density uncertainty) and uncertainties in the future use of the vault: such as: new equipment designs, patient loads, prescription methods (e.g., hypofractionation) and delivery methods. Given that these uncertainties are already considered, applying excessive conservatism in IMRT workload calculations could lead to overly conservative shielding designs, resulting in unnecessary and costly construction measures. Therefore, achieving accurate calculations to determine the optimal shielding thickness is essential, and this study focuses on providing data for precise calculations to support such accuracy. Although some studies provide general IMRT values for different techniques and LINACs, including NCRP 151 report,^3^, ^5^ the wide range of these values makes it challenging to perform accurate radiation shielding evaluations without precise data.

To prevent such confusions, it is essential to base analyses on accurate treatment data and to secure large‐scale, meaningful medical datasets. In the past, there were challenges associated with the extraction of DICOM plan files for numerous cases, along with the cumbersome task of re‐extracting them with each update.^9^ However, current advancements allow real‐time access to patient treatment records, enabling the exclusion of unnecessary parameters from DICOM plan files, thereby optimizing file size and facilitating the efficient export of multiple cases. In this study, we quickly retrieved the parameters necessary for calculating the IMRT factor and were able to easily evaluate the IMRT factor based on the MUs used in actual treatments by utilizing AURA. This streamlined process enables the periodic and efficient collection of radiation safety‐related parameters, contributing to enhanced radiation safety monitoring.

Our results showed that the IMRT factor for the Halcyon was high in fixed‐gantry IMRT techniques. VMAT uses fewer, larger segments while continuously rotating, covering larger areas with a single exposure. Conversely, fixed‐gantry IMRT uses many small segments from fixed angles for precise radiation control. Larger segments result in higher MU efficiency, leading to lower IMRT factors in Halcyon equipment for general VMAT techniques.^10^ The number of SABR treatment plans in this study was insufficient. However, with the increasing adoption of SABR treatments, further studies will acquire more data, enabling a more accurate assessment of IMRT factors for SABR. The number of breast plan cases was significantly higher compared to other plans, making clear comparisons between treatment sites challenging. Therefore, when comparing the median IMRT factor values across treatment sites in Figure 2, no prominent features were observed, regardless of whether the site was localized or extensive. Considering the possibility of including specific cases per patient, mean values and standard deviations were calculated, including all outliers, to ensure transparency and completeness of the analysis. Boost plans and reduced‐field cases, which could introduce confounding variability due to their unique characteristics, were excluded from this study.

Although an increase in the prescription dose requires precise and uniform control of the radiation beam to the target area, which can complicate IMRT treatment planning,^7^, ^8^ this does not necessarily mean that the IMRT factor will increase. Additionally, the SABR technique delivers an extremely high dose to the target area than VMAT and IMRT.^9^, ^10^, ^11^ Therefore, it is used to treat relatively small areas, and MU usage may be increased to achieve a uniform dose distribution; this characteristic does not contribute to an increase in the IMRT factor.

Several studies have reported that Halcyon tends to use more MUs compared to other equipment when planning for the same treatment site. This is due to the characteristics of the 6 MV flattening filter‐free beam, where a relatively lower PDD requires more MUs to deliver the same dose at the reference depth.^11^ Due to these characteristics, prominent radiation shielding literature suggests that the Halcyon exhibits an IMRT factor of approximately 10, which is roughly twice as high as that of the TrueBeam.^5^ However, in our study, after comparing multiple VMAT plans, while Halcyon generally showed a tendency to use higher MUs compared to the other two LINACs, the actual values remained within a comparable range and did not demonstrate large deviations, such as being two times higher than those of TrueBeam. If the IMRT factor of the Halcyon, calculated as approximately 2.82 ± 0.32 in this study, is compared with the IMRT factor of 10 suggested in shielding literature, the IMRT workload would increase by a factor of approximately 3.6. Accordingly, the required shielding thickness would increase by approximately 1.6 times, based on calculations using the tenth‐value layer formalism.

In some treatment plans, the IMRT factor approached or exceeded 10. These outlier values should be carefully considered from the perspective of the ALARA (As Low As Reasonably Achievable) principle, and they support the continued validity of conservative shielding designs that account for worst‐case scenarios. However, most clinical plans demonstrated lower IMRT factors, and the use of mean values for workload estimation may offer a more practical and realistic foundation for shielding design. In particular, average values derived from quantitative analysis of actual clinical data can serve as a reasonable compromise that satisfies the ALARA principle while avoiding excessive conservatism and unnecessary resource consumption. Therefore, this study proposes that, while acknowledging the existence of outliers, a mean‐value‐based approach grounded in real clinical data could serve as a viable and updated reference for shielding design.

The consistent variation in the IMRT factor across different LINACs can be attributed to the design and operation of the SX2 MLC on the Halcyon, the Millennium 120 MLC on the VitalBeam, and the High‐Density 120 MLC on the TrueBeam.^12^ The Millennium 120 MLC and High‐Density 120 MLC have widths of 0.5 and 0.25 cm, respectively. The current SX2 MLC is an upgraded version of the previous SX1, with both featuring dual‐layer stacked and staggered configurations.^13^ Additionally, the proximal MLC acts as a leaf tracking system to effectively prevent leakage and is known for its efficiency in dynamically flattening beams, outperforming the SX1 MLC in creating flattened beam plans. Therefore, while the SX2 MLC has the same resolution as the Millennium 120 MLC, it has lower interleaf transmission. Although the High‐Density 120 MLC offers higher resolution, enabling more complex dose distributions with higher modulation,^14^ it may also increase leaf leakage.

## 5 CONCLUSIONS

This study offers a practical estimation of IMRT factors derived from actual clinical data across three LINAC systems, contributing to a more informed and balanced approach to radiation shielding design. While prior literature has reported a broad—and often conservative—range for the IMRT factor, our findings indicate that most clinical plans are situated within a narrower, more clinically applicable range. The use of mean values, as proposed in this study, reflects current treatment practices and supports the ALARA (As Low As Reasonably Achievable) principle, helping to avoid excessive conservatism while ensuring the safety of patients and staff. These insights may serve as a useful contemporary reference for secondary shielding calculations, particularly in light of the growing adoption of dynamic MLC‐based techniques such as VMAT in routine clinical workflows.

## AUTHOR CONTRIBUTIONS

*Study Conceptualization*: Chang Heon Choi, Sungkoo Cho, and Jungwon Kwak. *Investigation, resources, data curation*: Jin Jegal, Min‑Jae Park and Sang Hoon Jung. *Software, Data analysis*: Chang Heon Choi and Jin Jegal. *Data validation*: Chang Heon Choi and Jung‐in Kim. *Writing—original draft preparation, visualization*: Jin Jegal. *Writing—review and editing*: Chang Heon Choi, Sungkoo Cho, Jungwon Kwak, and Jung‐in Kim. *Supervision, project administration*: Chang Heon Choi.

## CONFLICT OF INTEREST STATEMENT

The authors declare no conflicts of interest.

## ETHIC STATEMENT

This study was approved by the institutional review Board (IRB) of Seoul national university hospital (IRB No. 2303‐094‐1412) and Asan medical center (IRB No. 2024‐0333).
